# A qualitative study of the experiences of interdisciplinary nurses during the COVID-19 outbreak following the announcement of the “Ten new guidelines” in China

**DOI:** 10.1186/s12912-024-01905-0

**Published:** 2024-04-16

**Authors:** Li-Li Huang, Wei-Fen Wang, Wei-Wen Hong, Xian-Dan Huang, Xian-Hua Guan

**Affiliations:** 1 Department of Emergency, Taizhou First People’s Hospital, Taizhou, Zhejiang China; 2 Department of General Surgery, Taizhou First People’s Hospital, Taizhou, Zhejiang China; 3 Department of Nursing Management, Taizhou Hospital of Zhejiang Province, Linhai, Zhejiang China; 4 Intensive Care Unit, Taizhou First People’s Hospital, No. 218, Hengjie Road, Huangyan District, Taizhou, Zhejiang Province 318020 P. R. China

**Keywords:** Ten new guidelines, COVID-19, Interdisciplinary nurses, Qualitative research, Experience

## Abstract

**Background:**

On December 7, 2022, the Joint Prevention and Control Mechanism of China’s State Council released the “Ten New Guidelines” to optimize the coronavirus disease 2019 (COVID-19) prevention policies further. This signaled a broader shift from “dynamic clearing” to “coexisting with the virus” nationwide.

**Objective:**

This study aims to examine the experiences and perspectives of interdisciplinary nurses during the COVID-19 outbreak in China after the implementation of the “Ten New Guidelines”. The goal is to understand the challenges faced by this unique nursing group and inform organizational support to bolster their well-being and resilience.

**Methods:**

Two tertiary hospitals in southeastern Zhejiang Province were selected, with interdisciplinary nurses chosen as subjects. A constructivist qualitative research approach was employed, using semi-structured face-to-face interviews. Research data were collected through interviews and analyzed using content analysis.

**Results:**

Fifteen interdisciplinary nurses were included in this study. The analysis revealed four main themes and nine sub-themes. The main themes were: (1) ineffective organizational support (inadequate organizational care, poor PPE, excessive workload), (2) physiological distress after contracting COVID-19 (extreme physical fatigue, leakage of urine due to severe coughing), (3) fear of being wrong (fear of being reprimanded in public, psychological anxiety), and (4) family responsibility anxiety (difficulty of loyalty and filial piety, obligations to their children).

**Conclusion:**

We provide new evidence that organizations must proactively address the support, training, and communication needs of staff, particularly interdisciplinary nurses, to supplement epidemic containment. This is also essential in helping mitigate the work-family conflicts such roles can create.

**Supplementary Information:**

The online version contains supplementary material available at 10.1186/s12912-024-01905-0.

## Implications for clinical practice

- Organizations should provide supportive norms, and instrumental, and emotional assistance to improve the mental health of interdisciplinary nurses through fostering a perception of positive organizational support.
- Training, peer interactions, supervisor guidance, and self-reflection can help interdisciplinary nurses overcome challenges from unfamiliar workflows and skill gaps.
- Encouraging interdisciplinary nurses to seek family support and manage their time efficiently, combined with supervisor attention to mental health and access to professional psychological services, can aid in balancing work and family duties.

## Introduction

Since January 30, 2020, the World Health Organization (WHO) has designated the COVID-19 outbreak as a public health emergency of international concern [1]. After the SARS-CoV-2 variant Omicron became the predominant epidemic strain, some countries implemented “living with the coronavirus” policies, lifting travel restrictions and allowing free travel [2]. with the release of the “Ten New Guidelines for COVID-19 Prevention” in December 2022, China has shifted from “dynamic clearing” to “coexisting with the virus” [3]. These guidelines prioritized optimizing medical resources, elderly care, vaccination, testing, treatment, emergency capabilities, public communication, international exchanges, economic activity, and local-level response precision against the pandemic [3] (see Appendix 1 for “Ten New Guidelines”). One week after the publication of the “Ten New Guidelines,” a notable surge in community-based COVID-19 infections placed unprecedented strain on healthcare institutions, primary facilities for admitting and treating COVID-19 individuals, particularly for interdisciplinary nurses who work across a range of specialties.

Interdisciplinary nursing emphasizes collaboration and shared responsibility among medical professionals, going beyond traditional disciplinary boundaries to provide comprehensive patient care [4, 5]. During the COVID-19 outbreak, many nurses were rapidly redistributed across hospital departments to meet urgent staffing needs. We define these interdisciplinary nurses as registered nurses reassigned from their usual non-critical specialties (e.g. outpatient units) to backfill frontline roles in emergency, intensive care, and infectious disease departments. Despite lacking previous training or experience in these areas, this unique nursing group applied multifaceted knowledge and coordinated care across specialties. Their diverse experiences bridging clinical domains during the pandemic response offers valuable insights into interdisciplinary nursing practices. In previous studies, interdisciplinary nurses were mostly found in chronic disease management, nursing home interdisciplinary care plan practice, and coordinating the creation of clinical specialist programme proposals [6–8].

During the COVID-19 pandemic, interdisciplinary nurses from all over China were mobilized to aid the outbreak response [9]. We paid special attention to nurses redeployed from non-critical specialties to backfill frontline roles in departments such as respiratory medicine and infectious diseases that they lacked previous exposure to [10]. Prior studies have shown these types of reassigned nurses face heightened stress when adapting to high-pressure environments and caring for highly infectious patients [11]. We note that cultural aspects influenced the deployment of interdisciplinary nurses in Chinese healthcare organisations in the context of the pandemic, such as stratified workplace dynamics and collectivist norms [12]. Whereas, organisational and individual professionalism factors influenced the deployment of interdisciplinary nurses during the COVID-19 outbreak in countries such as Canada and Scotland [13, 14]. However, the bulk of studies on nurses mandated to epidemic hotspots focused on those dispatched between hospitals rather than within the same healthcare system [9, 11]. Our analysis addresses this gap by elucidating the distinct support needs of these internally redeployed nurses transitioning to critical frontline duties, to assist healthcare organizations in preparing for future pandemic responses.

## Methods

### Design

Data collection was conducted via face-to-face or telephone interviews, with content analysis used for data interpretation [15]. This study is grounded in constructivism, which posits that knowledge and meaningful reality are constructed through interactions with others and the external world, developing within a social context [16]. Accordingly, the study employs a qualitative research approach based on constructivist principles.

### Settings

The research was carried out in two tertiary hospitals located in southeastern Zhejiang Province. One hospital has a capacity of 2,000 beds, while the other accommodates 1,200 beds.

### Participant recruitment

Purposeful sampling was employed to select eligible participants for this study, aiming to gather relevant and insightful information from a diverse group of individuals [17]. The criteria for selecting interdisciplinary nurses included: (1) Registered nurses reassigned from non-critical departments; (2) Deployment to frontline COVID-19 units between December 2022 and January 2023; (3) A minimum of 5 years of recent experience in their original non-acute department; (4) Provision of direct bedside care during COVID-19 deployment; (5) Willingness to participate in interviews about their deployment experience. Nurses were excluded from the study if they (1) held management positions or (2) had internships or rotations in respiratory, infection, emergency, or intensive care units.

### Sample size

The sample size for this study was determined by achieving concept saturation. This point is reached when interviews begin to reveal repetitive themes without any new distinct concepts emerging, indicating that the information gathered is sufficiently powerful and representative. In this study, saturation was attained after conducting interviews with 15 nurses. At this point, a consensus emerged among the coders about the conceptual categories, which encapsulated key shared experiences, signifying that no additional interviews were necessary to enhance the understanding of the subject matter [18].

### Ethical approval

Before the interviews, participants were thoroughly informed about the anonymity and confidentiality of their information. They were also briefed on the study’s methodology, objectives, and their right to withdraw at any time voluntarily. Participants provided informed consent by signing a statement confirming their understanding of these aspects. The study protocol, numbered 2022-KY009-01, received ethical approval from the Ethics Committee of Taizhou First People’s Hospital in Zhejiang Province. All procedures conducted in the study adhered to the principles outlined in the Declaration of Helsinki [19].

### Data generation

**Fig. 1:**
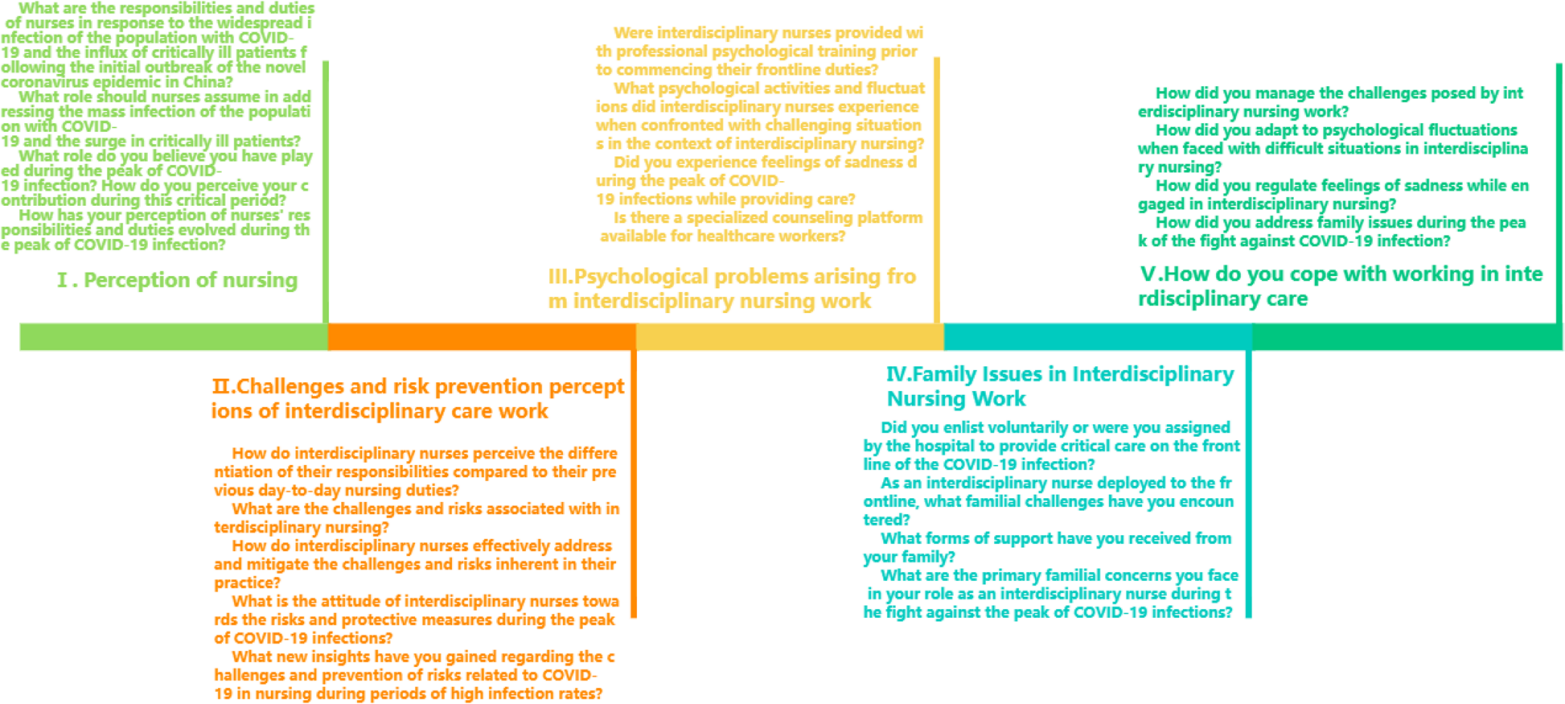
The interview topic guide

Individual interviews were conducted using a semi-structured interview guide from February 1st to February 20th, 2023. The guide, developed specifically for this study and informed by our own experiences in this unique context, included an outline with open-ended questions to facilitate the research dialogue [20] (see Fig. 1). The guide encompassed five main issues, each further detailed through four or five sub-issues: ‘Perceptions of nursing’, ‘Perceptions of challenges and risk prevention in interdisciplinary nursing’, ‘Psychological issues arising from interdisciplinary nursing’, ‘Family issues in interdisciplinary nursing’, and ‘How to cope with issues in interdisciplinary nursing’. Participants were asked additional survey and follow-up questions following the main and sub-questions.

Interviews took place at locations convenient for the participants, such as lounges or small meeting rooms, and were conducted in two formats: face-to-face and via telephone. All interviews were recorded with the interviewees’ consent and subsequently backed up. The average duration of the interviews was 51 min, ranging from 45 to 72 min. To ensure a comprehensive capture of the interdisciplinary nurses’ experiences, interviews were scheduled 3–20 days after their involvement in interdisciplinary support activities.

### Data analyses

Manifest content analysis was used for data analysis and interpretation. In this approach, researchers aim to provide a descriptive account of informants’ statements, closely aligning with the text to represent observable elements [21]. An inductive method was employed, identifying commonalities and differences in the data, moving from specific, tangible text to a broader conceptual level [22].

During the preparation phase, investigators, specifically L.L and W.F, read the transcribed interviews multiple times for data familiarization. They then identified meaningful units within the text [23], with each unit representing a set of words or sentences conveying a similar central concept or content [15]. In the organizing phase, these meaningful units were condensed into codes and merged into broader categories [15]. The research team then collaboratively determined the optimal interpretations of these categories and established final themes through triangulation.

Coding was initially conducted in Chinese and later translated into English for peer review by X.H [24]. The primary investigator, X.H, fluent in both Chinese and English, managed the translation process. Analysis reached saturation when the three coders observed repetitive responses from participants and no new themes emerged [25]. A thematic matrix was created using the coded data [25]. To highlight the varied expressions of ideas by participants and enhance the social relevance of the findings, quotations were selectively included for each theme, aiding in the delineation of theme boundaries [21].

## Findings

### Demographic characteristics of participants

Fifteen participants were selected based on the criterion of information repetition during the interviews, indicating that saturation was achieved with no new themes emerging [25]. The demographic composition of the 15 participants included 3 males and 12 females, with ages ranging from 21.0 to 45.0 years (mean 31.6 ± 6.8 years) and work experience averaging 9.0 years (range 7.0 to 15.0 years). Of these participants, 11 were married and 4 unmarried; 13 had tested positive for COVID-19 prior to their interdisciplinary support work; 3 had experience supporting the COVID-19 epidemic response in Wuhan or Shanghai. The participants were coded from TN1 to TN15. A summary of their demographic data is presented in Table 1.

**Table 1:** Presents a summary of the participants’ demographic data (*n* = 15)

No	Gender	Age (years)	Marital status	Education	Working experience (years)	Previous department	Support department	Interview time after support (days)	Infected with COVID-19 before support	Experience of supporting Wuhan/Shanghai
TN1	female	42	married	postgraduate	19	Nursing Department	ED	3	Y	N
TN2	female	32	married	undergraduate	9	General Surgery Department	ED	6	Y	Y
TN3	female	45	married	undergraduate	23	Interventional Room	ED	5	Y	N
TN4	female	28	married	undergraduate	8	Endoscopy Room	EICU	11	Y	N
TN5	male	23	unmarried	undergraduate	3	Endoscopy Room	EICU	11	Y	N
TN6	male	21	unmarried	college	2	Operating Room	EICU	15	Y	N
TN7	female	27	married	undergraduate	7	Operating Room	EICU	7	Y	N
TN8	female	37	married	undergraduate	15	Operating Room	EICU	15	N	Y
TN9	female	36	married	undergraduate	14	General Surgery Department	ICU	19	Y	Y
TN10	female	27	unmarried	college	7	Otolaryngology Department	ICU	17	N	N
TN11	female	23	unmarried	undergraduate	5	General Surgery Department	ICU	6	Y	N
TN12	female	32	married	undergraduate	10	Traditional Chinese Medicine Department	ICU	20	Y	N
TN13	female	37	married	undergraduate	16	Physical Examination Department	ICU	18	Y	N
TN14	female	34	married	undergraduate	13	Obstetrics and Gynecology Department	ICU	8	Y	N
TN15	male	30	married	undergraduate	8	Operating Room	PD	16	Y	N

*TN* Tnterdisciplinary Nurses, *ED* Emergency Department, *EICU* Emergency Intensive Care Unit, *ICU* Intensive Care Unit, *PD* Pneumology Department, *Y* Yes, *N* No

**Fig. 2:**
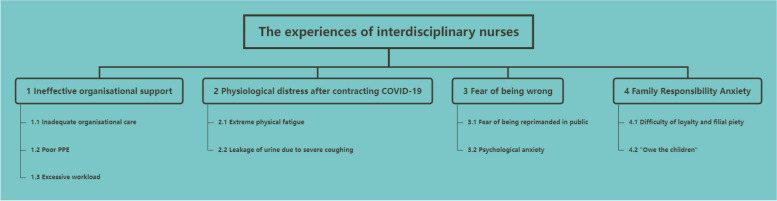
Thematic matrix

The analysis identified four main themes and nine subthemes: (1) “Ineffective Organizational Support,” encompassing inadequate organizational care, poor PPE, and excessive workload; (2) “Physiological Distress After Contracting COVID-19,” including extreme physical fatigue and urinary leakage due to severe coughing; (3) “Fear of Being Wrong,” characterized by the fear of public reprimand and psychological anxiety; and (4) “Family Responsibility Anxiety,” involving the challenges of loyalty and filial piety, and feelings of owing something to their children. The themes and subthemes are detailed in the thematic matrix (see Fig. 2).

### Ineffective organisational support

Based on an in-depth analysis of interdisciplinary nurses’ experiences during the COVID-19 outbreak, several inefficiencies in supporting these nurses within healthcare organizations were identified. This overarching theme was divided into three distinct sub-themes: insufficient organizational care, poor personal protective equipment (PPE), and excessive workload.

#### Inadequate organisational care

Mandatory dispatch, working while ill, and “ending inexplicably”. One nurse shared, “Without consulting me, I was told directly over the phone that I needed to report to work at the ICU tomorrow, making me feel passive in accepting the notice” (TN2). Another recounted, “I arrived at the respiratory unit and found out I had tested positive for COVID-19 antigen and was experiencing a high fever, cough, and body aches. Although I rested for 3 days and my fever subsided, I was immediately called back to work” (TN1). A third nurse expressed, “It ended so abruptly that I calmly returned to my old section and continued to be busy. However, no one knows how I suffered during the month I spent supporting others” (TN3).

#### Poor PPE

The rudimentary PPE and shortage of protective materials were identified as serious challenges. “We were only provided with surgical masks, and nurses working on external wards were not provided with protective clothing or isolation gowns. Disposable isolation gowns were only available in the isolation rooms for acute and critical patients” (TN5).

#### Excessive workload

Frequent night shifts, long overtime hours, and short recovery times illustrate the significant workload and excessive assignments faced by interdisciplinary nurses. “We worked on a 5-day night shift pattern, starting with the back night shift (0:00–8:00), followed by the front night shift (17:00–24:00), before taking a day off. My body didn’t have time to recover before starting another round of night shifts” (TN14). “Overtime has become the norm, at least an hour almost every day. It feels really tiring” (TN7).

### Physiological distress after contracting COVID-19

Physical challenges faced by interdisciplinary nurses returning to work after contracting new cases of COVID-19, referred to as Crown pneumonia, were significant. Two prominent sub-themes emerged: “extreme physical fatigue” and “leakage of urine due to severe coughing.”

#### Extreme physical fatigue

Respondents (*n* = 12) reported a significant decline in physical functioning, which was a major challenge. Symptoms of fatigue and weakness were common, leading to reduced immunity and loss of appetite. “During the support period, the slightest extra effort or brisk walk made me feel weak and breathless. I would be out of breath climbing stairs, and this lasted for several weeks” (TN8). Another nurse shared, “After contracting COVID-19, I couldn’t eat at all. Working for 8 h straight left me unable to stand up, feeling like I was about to collapse” (TN15).

#### Leakage of urine due to severe coughing

A number of female nurses (*n* = 8) experienced increased abdominal pressure due to violent coughing post-COVID-19 infection, leading to urinary incontinence. This caused significant distress, including forced work interruptions and body odor issues. “Continuous violent coughing led to persistent urinary incontinence, which I could only relieve by stopping my task and squatting down to reduce abdominal pressure” (TN12). Another nurse recounted, “As I was too busy to use the restroom, even the slightest cough would cause incontinence. I could smell the unpleasant odor myself throughout the day, which was very embarrassing” (TN7).

### Fear of being wrong

“Fear of being wrong” emerged as a significant theme in the data analysis, comprising two sub-themes: “fear of being publicly reprimanded” and “psychological anxiety.”

#### Fear of being reprimanded in public

Interviewees expressed emotional tension and intimidation stemming from a lack of knowledge about commonly used medicines in the support unit and proper care practices, leading to a pervasive fear of making mistakes and facing public blame. “I felt nervous every time I went to work, especially during the first week. I was worried that my doctor would find out I knew nothing” (TN4). Another nurse recounted, “When a patient’s blood glucose dropped to 2.1 mmol/L because insulin wasn’t stopped in time, the doctor was very angry and said, ‘You don’t even understand such simple common sense!’ I felt extremely ashamed and remorseful” (TN8).

#### Psychological anxiety

Inability to effectively participate in resuscitation due to unfamiliarity with the environment and item placement in the support unit caused worry, sleeplessness, and a severe lack of self-confidence. “As I was unfamiliar with the placement of items, I needed to keep asking others, which made me feel embarrassed and apologetic for disturbing them” (TN13). Another nurse shared, “Before going to bed, I would repeatedly think about the day’s work. If I got a call from the unit, I would feel nervous and unable to fall asleep for a long time” (TN11).

### Family responsibility anxiety

The interview data revealed “anxiety about family responsibilities” as another prominent theme, encompassing the sub-themes of “difficulty of loyalty and filial piety” and “owing the children.”

#### Difficulty of loyalty and filial piety

The increased workload due to the COVID-19 outbreak and the influx of patients into hospitals forced interdisciplinary nurses to choose between their professional duties and caring for their sick parents. This led to feelings of both mission and guilt. “Putting on our white coats means we’re on a mission and have to put our personal lives aside for a while,” shared one nurse (TN9). Another nurse, an only child, expressed, “Both of my parents were sick and hospitalized. I felt extremely guilty for sticking to my job and not being able to care for them under these circumstances” (TN10).

#### Owe the children

Interdisciplinary nurses treating COVID-19 patients often had to deliberately avoid close contact with their children after work, leading to an inability to provide care or help with homework. This resulted in a lack of emotional support they could offer as parents. “When I got home, I couldn’t have close contact with my child due to self-isolation, leaving me feeling deeply indebted to my child,” explained one nurse (TN13). Another recounted, “This month my son contracted COVID-19. Being deeply involved in work, I felt extremely lost not being able to be there for him when he needed me most” (TN16).

## Discussion

This study explored the experiences of 15 interdisciplinary nurses urgently redeployed to frontline COVID-19 units during the pandemic response in China. Key results reveal perceived gaps in organizational support alongside four critical impact areas tied to these temporary cross-department transitions. Specifically, analysis identified themes of insufficient workplace care and resources exacerbating staff burnout; severe post-COVID physical symptoms hindering work capacity; pervasive psychological fears of medical errors and public criticism over knowledge limitations; and pronounced family-related guilt due to inability to care for hospitalized relatives.

Previous research has shown that mental issues such as depression, anxiety, and insomnia disrupt the biological rhythms and work-life balance of interdisciplinary nurses [26]. However, higher levels of organizational support can protect the mental health of frontline workers during epidemic outbreaks [27]. Additionally, compared to prior studies on frontline nurses, findings spotlight several challenges uniquely amplified for participants rapidly transitioning between specialties, including intensified anxieties over unfamiliar protocols, debilitating fatigue, and role conflicts from simultaneously managing unfamiliar critical care duties and increased household obligations. Together these important discoveries contextualize real-world stresses interdisciplinary nursing teams confront when bridging across clinical domains, often with inadequate preparation or support. Discussion is warranted on organizational and training implications.

The International Council of Nurses (ICN) has developed a set of ethical guidelines for nurse reassignment, which can guide fair treatment and ethical decision-making during the reassignment process. These guidelines promote principles such as respecting autonomy, ensuring patient safety, and equitable workload distribution, thus improving professionalism and accountability in global nurse reassignment practices [28]. The guidelines provide guiding principles and standards to facilitate ethical decision-making and fairness among nurses during their reassignment to different roles or units within healthcare settings. By adhering to these guidelines, nurses can better manage ethical challenges that arise during the reassignment process and provide high-quality care for patients.

Another important finding is that unfamiliarity with work processes and inadequate skills can lead to anxiety among interdisciplinary nurses, and the risk of public criticism may exacerbate this anxiety. Similar findings have been documented in previous studies, particularly during pandemics, where nurses may experience anxiety due to unfamiliarity with infection control practices [29]. Our research results further deepen the understanding of this issue. This indicates the importance of providing appropriate training, guidance, and communication to alleviate anxiety and ensure that nurses are prepared in their new roles [30]. Interdisciplinary nurses also faced challenges in managing work pressures and family care during the outbreak, leading to feelings of guilt and responsibility anxiety. To address these concerns, nurses should seek support from family members and effectively manage their time, while higher authorities should prioritize their mental health and provide professional psychological support and assistance [31].

Situating these results within established interdisciplinary nursing theory allows richer illumination of participants’ experiences beyond the COVID-19 context alone. As interdepartmental transitions between specialized areas of care become increasingly prevalent amidst healthcare labor constraints, such frameworks help codify best practices for mitigating associated risks [32]. Core interdisciplinary principles around flexibility, collaboration, continuous learning, and coordinated care delivery can further contextualize barriers in participant adaptation [33]. Additionally, by connecting findings to fundamental interdisciplinary competencies in nursing, organizational and training deficiencies hampering participants may inform refined models, standards, and educational curricula for smoothing future redeployments. Thus grounding analysis of these vital frontline perspectives within the interdisciplinary nursing knowledge base serves both applied and theoretical advancement purposes. It transforms isolated discoveries into generalized, transportable insights strengthening the scaffolding undergirding sound interdisciplinary practice as provider roles rapidly evolve.

## Limitations

Several limitations are inherent in this study. Firstly, due to its qualitative and exploratory nature, establishing causality was not feasible. Secondly, the geographical homogeneity of the participants, all from the same region, could introduce directness effects, potentially impacting the study’s credibility and objectivity. Thirdly, no systematic differences were noted among participants working in the two hospitals. This observation implies that the limited sample size may not have introduced significant bias into our reported findings. Fourthly, while both hospitals adhered to the national-level pandemic policies set by Chinese health authorities, we did not gather detailed data to verify the absolute consistency in the specific approaches to nurse deployment. Lastly, considering the limitations of the quantifiable measures used in this study, conducting more comprehensive and extensive quantitative follow-up research in the future is recommended. Such studies would be valuable in further validating and enhancing the quality of the findings.

## Conclusion

Our study, with its specific focus on interdisciplinary nurses within hospitals, offers novel insights into the interplay between changes in infection control roles and psychosocial stress. We present new evidence underscoring the need for organizations to proactively address staff’s support, training, and communication needs, particularly interdisciplinary nurses. This approach is vital for augmenting epidemic containment efforts and alleviating the work-family conflicts that such roles often entail. Our findings highlight the importance of organizational support in ensuring the well-being and effectiveness of nurses as they navigate the complex demands of their roles during public health crises.

### Supplementary Information

**Supplementary Material 1.**

## Data Availability

The data that support the findings of this study are available on reasonable request from the corresponding author. The data are not publicly available due to privacy or ethical restrictions.
