# Supplementary material for: A qualitative study of the experiences of interdisciplinary nurses during the COVID-19 outbreak following the announcement of the “Ten new guidelines” in China
Source: BMC Nurs. 2024 Apr 16;23:244. doi: 10.1186/s12912-024-01905-0 (PMC11020795; doi:10.1186/s12912-024-01905-0)
Supplement: Supplementary file 1 — Supplementary Material 1. [file 12912_2024_1905_MOESM1_ESM.docx]

Appendix 1 the Ten New Guidelines

| Guidelines domain | Content of the domain |
| --- | --- |
| 1.Accurate scientific division of risk areas | High-risk areas should be delineated based on factors such as buildings, units, floors, and residents, and it is strictly prohibited to expand them to neighborhoods, communities, and streets (townships) and other regions. At the same time, various forms of temporary lockdown are prohibited. |
| 2.Optimization of nucleic acid testing | Universal nucleic acid testing will no longer be conducted based on administrative regions, further reducing the scope and frequency of nucleic acid testing. Antigen testing can be implemented according to the needs of epidemic prevention work. Personnel working in high-risk positions and individuals in high-risk areas need to undergo nucleic acid testing according to relevant regulations, while other individuals can choose to be tested based on their personal preferences. Except for special places such as nursing homes, welfare institutions, medical institutions, kindergartens, primary and secondary schools, there is no requirement to provide negative nucleic acid test certificates and health codes. Control measures for important institutions, large enterprises, and other specific places can be formulated by the local authorities. Cross-regional travelers no longer need to present negative nucleic acid test certificates and health codes, nor do they need to undergo landing tests. |
| 3.Optimization and adjustment of isolation methods | Infected individuals should be admitted for treatment based on the severity of their condition. Asymptomatic infected individuals and mild cases with suitable conditions for home isolation should generally undergo home isolation and can also choose centralized isolation voluntarily. Health monitoring should be strengthened during the home isolation period, and individuals with two consecutive nucleic acid test results with Ct values ≥35 on the 6th and 7th day of isolation can be released from isolation. Individuals with worsening symptoms should be promptly transferred to designated hospitals for treatment. Close contacts should undergo 5-day home isolation and can also choose centralized isolation, with isolation being lifted after a negative nucleic acid test on the 5th day. |
| 4.Implementation of "quick lockdown and quick release" in high-risk areas | Risk areas with no new infections for 5 consecutive days should be promptly released from lockdown. |
| 5.Guaranteeing the basic medication needs of the population | Local pharmacies must operate normally and must not be arbitrarily closed. The purchase of non-prescription drugs, such as antipyretics, cough suppressants, antiviral drugs, and cold medicines, should not be restricted for both online and offline transactions. |
| 6.Accelerating the vaccination of the elderly against the COVID-19 virus | Local authorities should adhere to the principle of accepting and vaccinating all eligible individuals, with a focus on increasing the vaccination rate among people aged 60-79 and accelerating the vaccination rate among those aged 80 and above. Measures such as setting up green channels for the elderly, temporary vaccination sites, and mobile vaccination vehicles should be implemented to optimize vaccination services. Training on contraindications for vaccination should be conducted to guide medical personnel in making informed decisions. Enhanced science popularization and mobilization of the entire society should encourage the elderly to actively participate in vaccination through incentive measures. |
| 7.Strengthening health assessment and classified management of key populations | Leverage the role of grassroots medical and health institutions as the "foundation" and family doctors as the "gatekeepers" to identify the elderly with cardiovascular diseases, chronic obstructive pulmonary disease, diabetes, chronic kidney disease, tumors, immunodeficiency, and their COVID-19 vaccine status within their jurisdiction, and implement graded and classified management. |
| 8.Ensuring the normal operation of society and basic medical services | Non-high-risk areas should not restrict personnel movement or suspend work, production, or business activities. Medical personnel, public security personnel, transportation logistics personnel, supermarket employees, essential supply workers, and utilities workers should be included in the "white list" management, and personal protection, vaccination, and health monitoring should be ensured. Normal medical services and basic supplies such as water, electricity, and heating should be guaranteed, efforts should be made to maintain the order of normal production work, and prompt solutions should be provided to address urgent issues raised by the public, meeting their basic needs during the epidemic response period. |
| 9.Strengthening safety measures related to the epidemic | It is strictly prohibited to block fire exits, unit doors, and entrance gates in any way, ensuring smooth access for medical treatment and emergency evacuation for the public. Promote the establishment of mechanisms for coordination between communities and specialized medical institutions to facilitate medical treatment for elderly individuals living alone, minors, pregnant women, disabled individuals, and patients with chronic diseases. Strengthen care and psychological counseling for individuals under lockdown, patients, and frontline workers. |
| 10.Further optimization of epidemic prevention and control in schools | All regions and schools must resolutely implement scientifically accurate prevention and control requirements. Schools without outbreaks should conduct regular offline teaching activities, and facilities such as supermarkets, canteens, sports venues, and libraries on campus should operate normally. Schools with outbreaks should accurately delineate risk areas, while ensuring the normal order of teaching and daily life outside the risk areas. |
